# A rare variant in *TRIOBP* linked to occupational noise exposure in Meniere disease

**DOI:** 10.1016/j.gendis.2025.101993

**Published:** 2025-12-19

**Authors:** Pablo Cruz-Granados, Giselle Bianco-Bortoletto, Yuzhong Zhang, Prathamesh T. Nadar-Ponniah, Kiana Bagheri-Loftabad, Edi Lúcia Sartorato, Ines Sanchez-Sellero, Andres Soto-Varela, Jose A. Lopez-Escamez

**Affiliations:** a Meniere Disease Neuroscience Research Program, Faculty of Medicine & Health, School of Medical Sciences, The Kolling Institute, University of Sydney, Sydney, New South Wales 2064, Australia; b Laboratory of Human Molecular Genetics, Center for Molecular Biology and Genetic Engineering - CBMEG, Programa de Pós Graduação em Ciências Médicas, Faculty of Medical Sciences, State University of Campinas-UNICAMP, Sao Paulo 13083-887, Brazil; c Department of Otorhinolaryngology-Head & Neck Surgery, West China Hospital of Sichuan University, Chengdu, Sichuan 610041, China; d Division of Toxicology, Department of Forensic Sciences, Pathology, Gynecology and Obstetrics, and Pediatrics, School of Medicine, Forensic Sciences Institute, Universidade de Santiago de Compostela, Santiago de Compostela 15782, Spain; e Division of Neurotology, Department of Otorhinolaryngology, Complexo Hospitalario Universitario de Santiago de Compostela, Santiago de Compostela 15706, Spain; f Department of Surgery and Medical-Surgical Specialities, School of Medicine, Universidade de Santiago de Compostela, Santiago de Compostela 15782, Spain; g Health Research Institute of Santiago (IDIS), Santiago de Compostela 15706, Spain; h Otology & Neurotology Group CTS495, Division of Otolaryngology, Department of Surgery, Instituto de Investigación Biosanitaria, ibs.GRANADA, Universidad de Granada, Granada 18016, Spain; i Sensorineural Pathology Programme, Centro de Investigación Biomédica en Red en Enfermedades Raras, CIBERER, Madrid 28029, Spain; j Ear Science Institute Australia, Nedlands, Western Australia 6008, Australia

Meniere disease (MD) is a lifelong disease characterised by episodes of vertigo, sensorineural hearing loss (SNHL) and tinnitus, and is associated with the accumulation of endolymph in the cochlear duct. Familial clustering and exome sequencing studies in multi-case families have provided evidence to associate ∼15–20 genes with MD.^1^

Noise-induced hearing loss can result in histopathological damage accompanied by acute inflammatory responses in cochlear structures, including the stria vascularis and the stereocilia of hair cells within the organ of Corti.^2^

The *TRIOBP* gene encodes TRIO and F-actin-binding protein, a structural protein found in several organs across the body, with isoforms 4 and 5 found primarily at the base of the hair cell stereocilia. Mutations in *TRIOBP* are associated with non-syndromic recessive SNHL (DFNB28) across different populations. The *TRIOBP*-encoded protein isoform 6 is the longest isoform found in humans, but its function is not well known since *TRIOBP*-6 is not expressed in murine species.^3^

To explore the link between genetic variation and environmental noise in MD, we retrieved clinical data from 77 Spanish individuals diagnosed with MD.^4^ Occupational noise and vibration exposure was classified based on job titles according to the International Standard Classification of Occupations (ISCO-08) and Spain's Social Security National Institute guide. The participants were categorised as exposed (*n* = 39) or non-exposed (*n* = 38) to noise and/or vibrations.

We obtained whole exome sequencing data from 17 MD individuals that were categorised as exposed to occupational noise and 17 MD categorised as not exposed (see *Material and Methods* in Appendix A -Supplementary Data). We found six rare missense variants in *CENPJ*, *PLEKHA7*, *SLC41A3*, *PRRC2C* and *NEURL4* (Table S1 and S2), and an ultrarare missense variant in the *TRIOBP* gene (chr22:37769343C>T, p.R2273C, AF = 3.22 × 10^−5^). The *TRIOBP* variant was found in 2/17 (11%) MD individuals in the noise exposed cohort, had a very low frequency in the Non-Finish European (NFE) population (odds ratio = 1846.76 [203.82–8192], adjusted *P*-value = 3.39 × 10^−5^), and was classified as Variant of Uncertain Significance according to the Hearing Loss-specific American College of Medical Genetics criteria (Fig. 1A).^k^ Notably, a variant in the *LGALS8-AS1* lncRNA found in the same two individuals with the *TRIOBP* variant was not observed in the MD noise-free cohort (Table S3).

**Figure 1:**
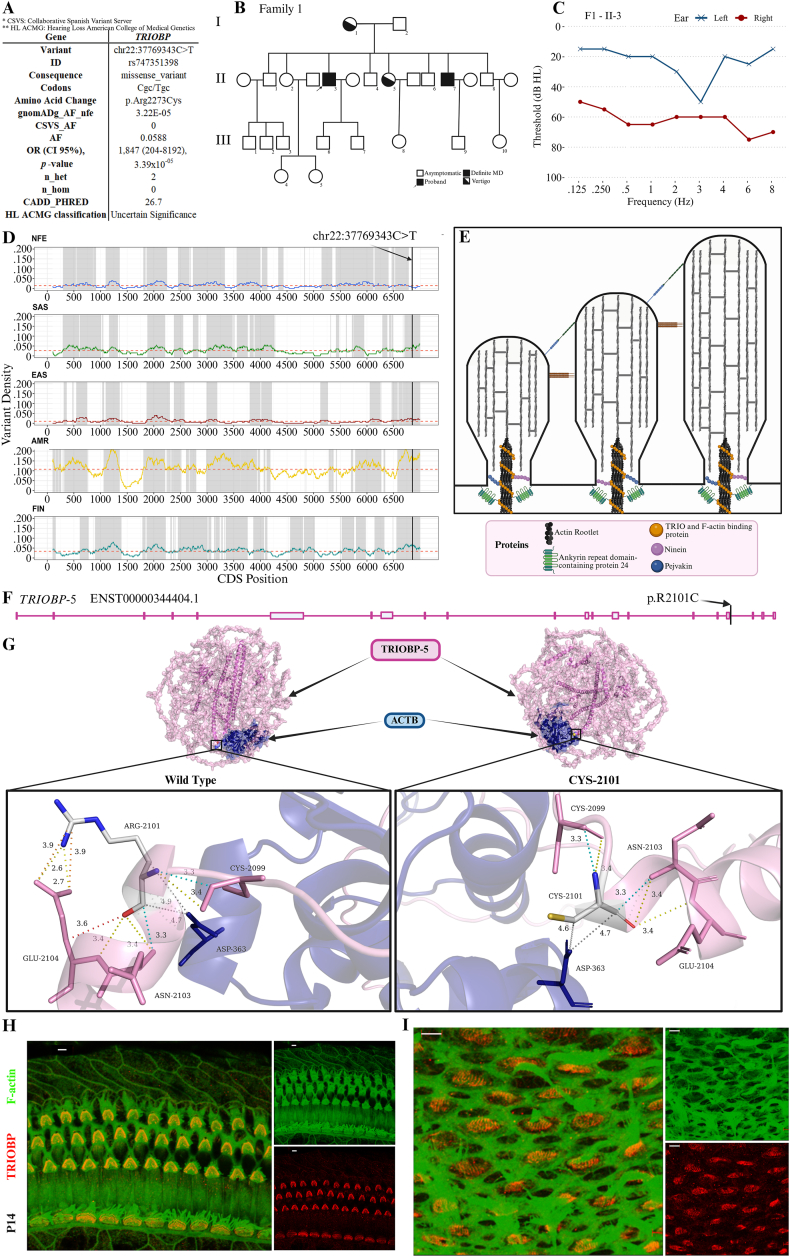
An ultra-rare *TRIOBP* variant disrupts stereocilia rootlet architecture and confers susceptibility to noise-induced hearing loss in Meniere disease. **(A)** Ultra-rare variant chr22:37769343C>T in the *TRIOBP* gene found in the occupational noise exposure cohort. **(B)** Pedigree of Family 1 with the *TRIOBP* chr22:37769343C>T variant. **(C)** Proband II-3 air conducted hearing threshold audiogram with bilateral SNHL. **(D)** Low-density (white) and high-density (grey) areas in *TRIOBP* CDS across different populations. **(E)***TRIOBP*-5 links F-actin proteins in the stereocilia rootlets to add rigidity to the structure. **(F)***TRIOBP*-5 ENST00000344404 transcripts. **(G)** Wild type (left) and mutant (right) TRIOBP-5 and ACTB interaction model, highlighting the atomic interaction between wild type (left) and mutant (right) TRIOBP-5 p.2101 and ACTB p.D363. **(H)** Postnatal day 14 (P14) mouse cochlear inner hair cells (IHCs) and outer hair cells (OHCs) stained for F-actin (green) and TRIOBP (red). Magnified overlay images of the stereocilia layer showing TRIOBP localisation at the base of the hair bundles. TRIOBP follows the V-shape labelling in OHC stereocilia. Scale bar: 5 μm. **(I)** P14 mouse utricle showing vestibular hair cells and supporting cells. Stereocilia layer of vestibular hair cells with TRIOBP and F-actin expression. TRIOBP labelling follows a linear distribution, contributing to the stereocilia rootlet of the vestibular HC. Scale bar: 5 μm.

We retrieved previously published whole genome bisulphite sequencing and single-cell ATACseq data (Table S5 and S6) and identified a significantly down-regulated *TRIOBP* 3’ UTR region on chr22:37696386–37697412 (fold change = −1.50; adjusted *P*-value = 2.97 × 10^−4^). Additionally, we found two significantly down-regulated UTR regions in the *RFX3* transcription factor that regulates *TRIOBP* expression (Table S4).

Patient 1, a man in his 70s with family history of MD (Fig. 1B), developed episodic vertigo and right sided pantonal SNHL in his late 40s, without any autoimmune or headache comorbidities (Fig. 1C). His brother, in his early 60s, was also diagnosed with bilateral MD and had a history of migraine and Tumarkin otolithic crisis (Fig. S1). Both siblings carried the *TRIOBP* chr22:37769343C>T variant.

Patient 2, a man in his mid-80s with a long-standing history of left-sided SNHL and episodic vertigo (Fig. S2), also carried the same variant. He had no family history or other relevant MD-associated conditions.

To explore the population-level missense variant distribution, we analysed the *TRIOBP* coding sequence (CDS) and defined high-density (HDR) and low-density regions (LDR) of the variants. Notably, the chr22:37769343C>T variant (CDS position 6817) lies in a LDR in the NFE population, but maps to HDRs in South Asian, East Asian, Admixed American, and Finnish populations (Fig. 1D), highlighting potential population-specific constraints in the NFE.

The TRIO and F-actin-binding protein is located at the base of the stereocilia, around the actin rootlet, providing stability together with other proteins such as ankyrin repeat domain-containing protein 24, ninein, or pejvakin (Fig. 1E).

We generated a pathogenicity heatmap from AlphaMissense information for the canonical sequence p.R2273C variant and found it in a benign site (Fig. S3). No splice sites were detected for the *TRIOBP* variant using *Pangolin* or *SpliceAI*. However, *HSF Pro* found a new donor splice site generated around the chr22:37769343C>T variant (Table S7), and several exonic splicing enhancers and silencers that were associated with the *TRIOBP* canonical and alternative transcripts (Table S8).

Homology modelling using SWISS-MODEL was performed on the canonical transcript and transcript ENST00000344404.10, which includes the p.R2273C and p.R2101C variants, respectively (Fig. 1F; Fig. S4). Computational-based structural analysis revealed two polar interactions between the TRIOBP-5 residue p.2101 and the β-actin residue p.363 (Fig. 1G). In the monomeric model, the p.R2101C mutation shortened one of these contacts. Computational stability analysis predicted a delta change (ΔΔG) of 0.72 kcalmol^–1^, suggesting a mild destabilising effect. Variant p.R2273C does not interact with ACTB (Fig. S5 and S6).

In the dimeric model of TRIOBP-5 docked with β-actin, the mutation caused notable changes in the interaction profiles. Both wild-type and mutant residues gained and lost contacts relative to the monomeric model. Crucially, the interaction between TRIOBP-5 (p.2101) and β-actin (p.363) was observed only in one TRIOBP-5 chain (chain C), not both (chains A and C), indicating altered binding symmetry. DynaMut2 predicted that the arginine-to-cysteine substitution results in a stabilising ΔΔG of 0.78 kcalmol^–1^ in chain A and 0.67 kcalmol^–1^ in chain C of the molecule (Fig. S7 and S8). Furthermore, computational modelling of TRIOBP-5 and -6 did not predict docking of arginine-to-cysteine variant with F-actin (Fig. S9–S12).

We further investigated TRIOBP expression in the auditory system. Immunofluorescence analysis of the inner ears of postnatal day 14 (P14) mice revealed TRIOBP localisation at the rootlets of inner hair cells (IHCs) and outer hair cells (OHCs), as well as in the vestibular hair cells of the utricle (Fig. 1H, I). Staining with phalloidin showed that the *TRIOBP*-encoded protein was concentrated near the apical surface at the stereocilia base. Additionally, ninein (encoded by the *NIN* gene) (Fig. S13) was expressed in the stereocilia rootlets of cochlear hair cells, but the protein model did not show a direct interaction with TRIOBP-5; however, it did with TRIOBP-6 (Fig. S14 and S15).

Most genes previously linked to MD, such as *OTOG*, *MYO7A* and *TECTA*, encode proteins localised to structures like the stereocilia apical crowns, which link the hair cell stereocilia to the tectorial membrane, horizontal top connectors, and the tectorial membrane.^1^ Evidence from rare variant analyses in sequencing studies in familial and sporadic MD and auditory brain responses from knockout models support the *NIN* and *ANKRD24* genes as novel candidate genes for MD.^5^ Taking these insights together with the *TRIOBP* findings reported here, we hypothesise that cytoskeleton proteins in hair cell stereocilia rootlets are also linked to MD. These observations support the hypothesis that disruption of the structural integrity of stereocilia contributes to the audiovestibular phenotype observed in MD. Our study extends the current understanding by characterising the protein composition of the hair cell rootlet and proposing *TRIOBP*, a gene essential for maintaining stereocilia rootlet structure, as a novel candidate gene for MD, particularly in the context of noise-induced hearing loss. These findings are exploratory, based on two variant carriers among 17 noise-exposed cases, and further studies are needed to validate the functional impact of this variant and to explore gene–environment interactions, including noise exposure in the pathophysiology of MD. A potential knock-in mouse model harbouring the p.R2273C mutation, combined with noise exposure, could be used to reproduce the human MD phenotype.

To conclude, an ultra-rare variant in the *TRIOBP* gene was found in several unrelated MD individuals with a history of occupational noise exposure. Computational modelling revealed that the variant modified the atomic bonds between the *TRIOBP-* and *ACTB-*encoded proteins, leading to increased stability of the dimer. These findings support that rare mutations in *TRIOBP*, in combination with environmental factors such as noise exposure, may contribute to changing the stiffness of hair cell stereocilia and the development of MD.

## CRediT authorship contribution statement

**Pablo Cruz-Granados:** Writing – review & editing, Writing – original draft, Visualization, Formal analysis, Data curation. **Giselle Bianco-Bortoletto:** Writing – review & editing, Writing – original draft, Visualization, Formal analysis. **Yuzhong Zhang:** Writing – review & editing, Writing – original draft, Visualization, Formal analysis. **Prathamesh T. Nadar-Ponniah:** Writing – review & editing, Writing – original draft, Visualization, Formal analysis. **Kiana Bagheri-Loftabad:** Writing – review & editing, Data curation. **Edi Lúcia Sartorato:** Writing – review & editing. **Ines Sanchez-Sellero:** Writing – review & editing. **Andres Soto-Varela:** Writing – review & editing. **Jose A. Lopez-Escamez:** Writing – review & editing, Writing – original draft, Funding acquisition, Conceptualization.

## Ethics declaration

The Human Ethics Research Committee (2023/HE000199) from The University of Sydney approved the protocol for this study. A written inform consent was obtained from all participants to donate blood samples, extract DNA for its sequencing and perform genetic analyses. This work was performed under the standards of the Declaration of Helsinki. The Animal Ethics Research Committee (approval no. 2023/2388) from The University of Sydney and the Animal Ethical and Welfare (approval no. 20240219057) from West China Hospital of Sichuan University evaluated and approved the protocol for this study.

## Data availability

The *TRIOBP* chr22:37769343C>T variant has been deposited in ClinVar under accession number SCV006304769.

## Funding

This research was funded by K7013-B3414G Grant from 10.13039/501100001774The University of Sydney.

## Conflict of interests

All the authors declare that there is no conflict of interests.

## Footnotes

k Created in BioRender. https://www.biorender.com/69ledgp.
