# Supplementary material for: A rare variant in TRIOBP linked to occupational noise exposure in Meniere disease
Source: Genes Dis. 2025 Dec 19;13(6):101993. doi: 10.1016/j.gendis.2025.101993 (PMC13380108; doi:10.1016/j.gendis.2025.101993)
Supplement: Multimedia component 1 [file mmc1.pdf]

Supplementary Data – Table of Contents

Material and Methods 4

Table S1.- Rare variants in *TRIOBP*, *CENPJ*, *PLEKHA7* and *SLC41A3* genes found in the occupational noise exposure cohort. 14

Table S2.- Rare variants in *PPRC2C* and *NEURL4* genes found in the occupational noise exposure cohort. 14

Table S3.- LGALS8-AS1 lncRNA chr1:236524482A>G variant in the MD noise cohort. 15

Table S4.- Differentially accessible peaks in *RFX3* genes on pseudo-Bulk ATACseq MD patients. 15

Table S5.- *TRIOBP* differentially methylated CpG sites in MD patients. 16

Table S6.- Differentially accessible peaks in *TRIOBP* genes on pseudo-Bulk ATACseq MD patients. 16

Table S7.- Splice site prediction for *TRIOBP* chr22:37769343C>T variant in transcripts ENST00000403663 and ENST00000644935. 16

Table S8.- Creation and deletion of Exonic Splicing Enhancers (ESE) and Exonic Splicing Silencers (ESS) regulatory elements in *TRIOBP* chr22:37769343C>T variant 16

Figure S1.- Family 1 - member II-7 air conducted hearing threshold audiogram with bilateral SNHL. Left ear represented in blue, right in red 17

Figure S2.- Meniere Disease individual 3 with occupational noise exposure. Audiogram showing air-conducted pure tone hearing thresholds in their seventh decade of life with bilateral high-frequency hearing loss and SNHL involving all frequencies in the left ear. Right ear is represented in red, left ear in blue. 18

Figure S3.- *TRIOBP* p.R2273C (chr22:37769343C>T) pathogenicity heatmap. AlphaMissense scores *TRIOBP* variant p.R2273C as likely being with a value of 0.239. 19

Figure S4.- A) Wild type (left) and mutant (right) TRIOBP-5 protein model, zooming in at the atomic interactions of TRIOBP-5 p.2101. B) Wild type (left) and mutant (right) TRIOBP-6 protein model, zooming in at the atomic interactions of TRIOBP-6 p.2273. 20

Figure S5.- Docked model between wt *TRIOBP*-6 (orange) and *ACTB* (blue). Variant p.2273R was not predicted to interact with *ACTB*.. 21

Figure S6.- Docked model between mt *TRIOBP*-6 (orange) and *ACTB* (blue). Variant p.2273C was not predicted to interact with *ACTB*.. 22

Figure S7.- A) Wild type *TRIOBP*-5 dimer docked model to *ACTB* chain C. B) Polar contacts of wt *TRIOBP*-5 dimer docked to *ACTB* chain A. B) Polar contacts of wt *TRIOBP*-5 dimer docked to *ACTB* chain C. 23

Figure S8.- A) Mutant *TRIOBP*-5 dimer docked model to *ACTB* chain C. B) Polar contacts of mutant *TRIOBP*-5 dimer docked to *ACTB* chain A. B) Polar contacts of mutant *TRIOBP*-5 dimer docked to *ACTB* chain C.. 24

Figure S9.- Docked model between wt *TRIOBP*-6 (orange) and F-Actin (magenta, cyan, blue and red). Variant p.2273R was not predicted to interact with F-Actin.. 25

Figure S10.- Docked model between mt *TRIOBP*-6 (orange) and F-Actin (magenta, cyan, blue and red). Variant p.2273C was not predicted to interact with F-Actin.. 26

Figure S11.- Docked model between wt *TRIOBP*-5 (orange) and F-Actin (magenta, cyan, blue and red). Variant p.2273R was not predicted to interact with F-Actin.. 27

Figure S12.- Docked model between mt *TRIOBP*-5 (orange) and F-Actin (magenta, cyan, blue and red). Variant p.2273C was not predicted to interact with F-Actin.. 28

Figure S14.- Docked model between wt *TRIOBP*-6 (orange) and *NIN* (cyan). Wt p.2273R was not predicted to interact with *NIN*.. 30

Figure S15.- Docked model between mt *TRIOBP*-6 (orange) and *NIN* (cyan). Variant p.2273C was not predicted to interact with *NIN*.. 31

Material and Methods

Human and Animal Ethics

The Human Ethics Research Committee (2023/HE000199) from The University of Sydney approved the protocol for this study. A written inform consent was obtained from all participants to donate blood samples, extract DNA for its sequencing and perform genetic analyses. This work was performed under the standards of the Declaration of Helsinki.

The Animal Ethics Research Committee (approval no. 2023/2388) from The University of Sydney and the Animal Ethical and Welfare (approval no. 20240219057) from West China Hospital of Sichuan University evaluated and approved the protocol for this study.

Patient Selection

Clinical data pertaining occupational noise exposure across a three-year study was retrieved from 77 individuals from Spain with diagnosed MD.[1] Participants were categorised as exposed to noise and/or vibrations (n = 39) or not exposed to noise and/or vibrations (n = 38). These risks were listed for their occupation (International Standard Classification of Occupations, 2008 (ISCO-08) in Spain's Social Security National Institute (SSNI) guide.

We obtained whole exome sequencing (WES) data from 17 MD individuals that were categorised as exposed to occupational noise and/or vibrations, and 17 MD categorised as not exposed.

Genomic Datasets

Previously published exome and genome sequencing data from MD participants were retrieved.[2,3] Gene burden analyses (GBA) were performed to generate a list of MD genes with excess of rare variants overlapping in these datasets.

Hearing loss associated genes were retrieved from the Deafness Variation Database.[4] In addition, we also retrieved single-cell RNA sequencing differentially expressed genes (DEG) found in the stria vascularis (SV), marginal cells (MC), intimidate cells (IMC) and basal cells (BC) of P30 mice.[5]

Finally, a list of genes from WES data associated with noise-induce hearing loss was retrieved and used to generate a list of NIHL genes for further analyses.[6]

Identification of Rare Variants in the Occupational Noise Cohort

Single nucleotide variants and short indels in the noise and non-noise cohorts were retained if their allelic frequency (AF) <0.05 in Non-Finnish European (NFE) from gnomAD and found at least in two out of the 17 (11.7%) individuals in their respective category.

We retrieved variants from the noise cohort that overlapped with the MD list, the DVD list, and the SV list; NIHL gene list was used as an internal control. The same variant extraction was done for the MD cohort without noise-exposure. The retrieved gene variants from the noise and non-noise exposed cohort were compared, and replicated variants were discarded.

Long non-coding RNA (lncRNA) variants were also extracted from individuals with rare variants in the noise cohort. LncRNAs that regulate the expression of candidate genes, as well as those that modulate the activity of co-expressed genes, were retained for further analyses.

Statistical Analysis

Allelic frequencies were obtained for the three variants sets and were compared to the AFs of the NFE reference dataset from gnomAD v4.1.0[7] to calculate the odds ratio (OR), 95% confidence interval (CI) and *p*-value adjusted using the Bonferroni correction.

Chromatin accessibility and Methylation Profiling of Candidate Genes

Epigenetic information from MD patients' peripheral blood mononuclear cells generated using Whole Genome Bisulphite Sequencing (WGBS)[8] and pseudo-bulk ATAC sequencing (ATACseq)[9] was retrieved form previously published studies. The WGBS dataset was used to determine differentially methylated regions, whereas the ATACseq dataset was used to determine chromatin accessibility regions. The ATACseq data was generated differential peak analysis of MD cases against a set of internal controls (HC) and external controls (EC).

Pathogenicity and Splice Site Prediction

Splice sites were predicted using *SpliceAI*[10] and *Pangolin*[11] for selected proteins using the canonical and relevant sequences. An upstream and downstream window consisting of 500-base pair (bp) from the variant position was used. In addition, Exonic Splicing Enhancer (ESE), Exonic Splicing Silencers (ESS), Intronic Splicing Enhancers (ISE) and Intronic Splicing Silencers (ISS) were predicted using *Human Splice Finder Pro* (HSF Pro).[12]

Pathogenicity scores were predicted using CADD score, PolyPhen2[13] and REVEL,[14] and pathogenicity heatmaps were generated for missense variants in canonical sequences from AlphaMissese[15,16] pathogenicity predictions. GnomAD v4.1.0 *Z*-scores were obtained to evaluate constraint regions around the variant of interest. The Variant Interpretation Platform for Genetic Hearing Loss[17] was used to classify the pathogenicity of the variants using the American College of Medical Genetics and Genomics (ACMG) criteria adjusted to Genetic Hearing Loss.[18]

Constraint regions with an overload of missense variants in genes of interest were identified by calculating the density of variants in the coding sequence (CDS). A sliding window consisting of 100 bp upstream and 100 bp downstream for the chosen mutations was created. The missense variants were retrieved from gnomAD v.2.1 database[19] for each population. Estimation of the high-density threshold was calculated based on the expected number of missense variants for each population defined in gnomAD v.2.1. Regions were classified as low-density if the computed density of the window was below the predicted density for each population.

Protein Modelling

Wild type (wt) sequences of interest were retrieved from UniProt[20] and PDB files were obtained from AlphaFold Protein Structure Database[16] and OtoProtein2 Database.[21] Atomic interactions of the wild type models were examined using ERRAT[22] from Saves v6.1 server (https://saves.mbi.ucla.edu/). For proteins without experimentally resolved structures or models with a ERRAT score < 45, models were obtained using AlphaFold 3[23] (*ab initio*) and Swiss-Model[24] (by homology).

Mutated proteins were generated by homology from wild type templates using MODELLER v10.6[25] and Swiss-Model. MODELLER bult-in quality controls (DOPE and GA341) were used to determine

suitability of the model. The chosen model had the lowest DOPE score and a GA341 score close to one. Swiss-Model predicts protein 3D structures by homology modelling by aligning target sequences with reference templates from their database. The selection of the optimal model is based on QMEAN scoring, which is a combination of geometry, solvation and agreement with expected properties. The reliability of the models is further validated through the implementation of GMQE (Global Model Quality Estimate) and local *Z*-scores, which identify and highlight any unreliable regions.

External quality checks were performed on mutated proteins using ERRAT, WHATCHECK (https://swift.cmbi.umcn.nl/gv/whatcheck/index.html) and PROCHECK[26] validation algorithms from Saves v6.1 server. WHATCHECK and PROCHECK were employed for assessing stereochemical quality of the models.

The chosen wt and mutated protein models were analysed for structural changes using PyMOL (*Schrodinger*, LLC. 2010. The PyMOL Molecular Graphics System, Version 3.0.). Finally, DynaMut2[27] was used for predicting protein stability on the mutant proteins.

Protein-Protein Interactions

Prediction of functional partners was performed using STRING v12[28] on the selected proteins, just using from "curated databases" and "experimentally determined" options. BioGRID v4.4 (https://wiki.thebiogrid.org/, accessed on the 10th of February 2025) and HINT[29] (https://hint.yulab.org/, accessed on the 27th of February 2025) were used to determined experimentally protein-protein interactions for selected proteins. The gEAR portal[30] *Human inner ear organoids - scRNAseq and snRNAseq dataset*[31] was used to determine expression in inner ear of predicted interactions with selected proteins. Proteins that expressed a fold change >1 in hair cells were retained for further analyses.

The International Mouse Phenotyping Consortium (IMPC)[32,33] was used to determine if gene knockout of functional patterns, mice developed hearing loss or vestibular dysfunction.

Molecular Docking

Prediction of interaction sites between the proteins of interest and functional partners was performed using DockNet.[34] ClusPro[35] was used to assess the molecular docking of selected PDB structures. The quality of the model is assessed via consensus clustering (reproducibility across scoring functions) and interface-specific metrics such as pairwise residue contacts (e.g., CAPRI criteria). The selection of the optimal model was based on cluster population (highest-density centres) and balanced scoring (electrostatics + van der Waals + decoy discrimination).[36] DynaMut2 was used to evaluate protein stability in the mutant proteins of interest once docked to functional partners.

Histology and Immunolocalisation

Mouse inner ear samples were prepared for whole-mount immunofluorescence as follows. After deep anaesthesia and decapitation, the inner ears were dissected and fixed in 4% paraformaldehyde (PFA) (Cat#: BL539A, Biosharp) at 4°C overnight.

The cochlear basilar membrane and vestibular macula were carefully dissected under a stereomicroscope, fixed for 30 minutes, and then decalcified in 10% EDTA for an additional 30 minutes. Tissues were permeabilised and blocked in a solution containing 1% Triton X-100 (Cat#: 1139ML100, BioFroxx) and 5% bovine serum albumin (BSA) (Cat#: SRE0098-10G, Sigma). Samples were then incubated overnight at 4°C with rabbit anti-ninein primary antibody (1:500; Cat#: 13007-1-AP, Proteintech) or *TRIOBP* antibody (1:500, Proteintech 161241-AP) diluted in a solution of 0.1% Triton X-100 and 5% BSA. The following day, samples were washed three times with 1× PBS containing 0.1% Triton X-100, then incubated for 2 hours at room temperature with secondary antibody (Donkey anti-Rabbit IgG; Cat#: A32795, Invitrogen) , Goat anti rabbit Alexa Fluor™ 594 (Cat # ab150080) and Alexa Fluor™ 555 Phalloidin (Cat#: A34055, Invitrogen), Phalloidin-iFluor 488 (Cat # ab176753). After additional washing with 1× PBS containing 0.1% Triton X-100, nuclei were counterstained with DAPI (Cat#: C1006, Beyotime). Finally, samples were mounted either using anti-fade fluorescence mounting medium (Cat#: AB104135, Abcam) or Prolong Gold antifade mounting medium (Cat #P36935) and imaged using a Leica Stellaris 5 confocal microscope.

References

1. Sánchez-Sellero I, Soto-Varela A. Relationship Between Occupational Exposure to Noise and Vibrations and Vertigo: A Prospective Case-Control Study. *J Clin Med*. 2024;13(22). doi:10.3390/jcm13226650

2. Parra-Perez AM, Gallego-Martinez A, Escalera-Balsera A, Robles-Bolivar P, Perez-Carpena P, Lopez-Escamez JA. Different contribution of missense and loss-of-function variants to the genetic structure of familial and sporadic Meniere disease. *MedComm (Beijing)*.

3. Fisch KM, Rosenthal SB, Mark A, et al. The genomic landscape of Ménière's disease: a path to endolymphatic hydrops. *BMC Genomics*. 2024;25(1):646. doi:10.1186/s12864-024-10552-3

4. Azaiez H, Booth KT, Ephraim SS, et al. Genomic Landscape and Mutational Signatures of Deafness-Associated Genes. *The American Journal of Human Genetics*. 2018;103(4):484-497. doi:10.1016/j.ajhg.2018.08.006

5. Thulasiram MR, Yamamoto R, Olszewski RT, et al. Molecular differences between young and mature stria vascularis from organotypic explants and transcriptomics. *iScience*. 2025;28(2):111832. doi:10.1016/j.isci.2025.111832

6. Fan B, Wang G, Liu G, Zhang X, Wu W. Whole-exome sequencing for screening noise-induced hearing loss susceptibility genes. *Acta Otolaryngol*. 2023;143(5):408-415. doi:10.1080/00016489.2023.2201287

7. Chen S, Francioli LC, Goodrich JK, et al. A genomic mutational constraint map using variation in 76,156 human genomes. *Nature*. 2024;625(7993):92-100. doi:10.1038/s41586-023-06045-0

8. Patil V, Cruz-Granados P, Cara FE, et al. Whole genome DNA methylation profiles define Meniere's disease subclusters. *J Mol Med*. Published online August 6, 2025. doi:10.1007/s00109-025-02581-6

9. Cruz-Granados P, Frejo L, Perez-Carpena P, et al. Multiomic-based immune response profiling in migraine, vestibular migraine and Meniere's disease. *Immunology*. 2024;173(4):768-779. doi:10.1111/imm.13863

10. Jaganathan K, Kyriazopoulou Panagiotopoulou S, McRae JF, et al. Predicting Splicing from Primary Sequence with Deep Learning. *Cell*. 2019;176(3):535-548.e24. doi:10.1016/j.cell.2018.12.015

11. Zeng T, Li YI. Predicting RNA splicing from DNA sequence using Pangolin. *Genome Biol*. 2022;23(1):103. doi:10.1186/s13059-022-02664-4

12. Desmet FO, Hamroun D, Lalande M, Collod-Béroud G, Claustres M, Béroud C. Human Splicing Finder: an online bioinformatics tool to predict splicing signals. *Nucleic Acids Res*. 2009;37(9):e67. doi:10.1093/nar/gkp215

13. Adzhubei IA, Schmidt S, Peshkin L, et al. A method and server for predicting damaging missense mutations. *Nat Methods*. 2010;7(4):248-249. doi:10.1038/nmeth0410-248

14. Ioannidis NM, Rothstein JH, Pejaver V, et al. REVEL: An Ensemble Method for Predicting the Pathogenicity of Rare Missense Variants. *The American Journal of Human Genetics*. 2016;99(4):877-885. doi:10.1016/j.ajhg.2016.08.016

15. Jumper J, Evans R, Pritzel A, et al. Highly accurate protein structure prediction with AlphaFold. *Nature*. 2021;596(7873):583-589. doi:10.1038/s41586-021-03819-2

16. Varadi M, Bertoni D, Magana P, et al. AlphaFold Protein Structure Database in 2024: providing structure coverage for over 214 million protein sequences. *Nucleic Acids Res*. 2024;52(D1):D368-D375. doi:10.1093/nar/gkad1011

17. Xiang J, Peng J, Baxter S, Peng Z. AutoPVS1: An automatic classification tool for PVS1 interpretation of null variants. *Hum Mutat*. 2020;41(9):1488-1498. doi:10.1002/humu.24051

18. Oza AM, DiStefano MT, Hemphill SE, et al. Expert specification of the ACMG/AMP variant interpretation guidelines for genetic hearing loss. *Hum Mutat*. 2018;39(11):1593-1613. doi:10.1002/humu.23630

19. Karczewski KJ, Francioli LC, Tiao G, et al. The mutational constraint spectrum quantified from variation in 141,456 humans. *Nature*. 2020;581(7809):434-443. doi:10.1038/s41586-020-2308-7

20. Bateman A, Martin MJ, Orchard S, et al. UniProt: the Universal Protein Knowledgebase in 2023. *Nucleic Acids Res*. 2023;51(D1):D523-D531. doi:10.1093/nar/gkac1052

21. Tollefson MR, Gogal RA, Weaver AM, et al. Assessing Variants of Uncertain Significance Implicated in Hearing Loss Using a Comprehensive Deafness Proteome. *Res Sq*. Published online February 1, 2023. doi:10.21203/rs.3.rs-2508462/v1

22. Colovos C, Yeates TO. Verification of protein structures: Patterns of nonbonded atomic interactions. *Protein Science*. 1993;2(9):1511-1519. doi:10.1002/pro.5560020916

23. Abramson J, Adler J, Dunger J, et al. Accurate structure prediction of biomolecular interactions with AlphaFold 3. *Nature*. 2024;630(8016):493-500. doi:10.1038/s41586-024-07487-w

24. Waterhouse A, Bertoni M, Bienert S, et al. SWISS-MODEL: homology modelling of protein structures and complexes. *Nucleic Acids Res*. 2018;46(W1):W296-W303. doi:10.1093/nar/gky427

25. Šali A, Blundell TL. Comparative Protein Modelling by Satisfaction of Spatial Restraints. *J Mol Biol*. 1993;234(3):779-815. doi:10.1006/jmbi.1993.1626

26. Laskowski RomanA, Rullmann JAntoonC, MacArthur MalcolmW, Kaptein R, Thornton JanetM. AQUA and PROCHECK-NMR: Programs for checking the quality of protein structures solved by NMR. *J Biomol NMR*. 1996;8(4). doi:10.1007/BF00228148

27. Rodrigues CHM, Pires DEV, Ascher DB. DynaMut2: Assessing changes in stability and flexibility upon single and multiple point missense mutations. *Protein Science*. 2021;30(1):60-69. doi:10.1002/pro.3942

28. Szklarczyk D, Kirsch R, Koutrouli M, et al. The STRING database in 2023: protein-protein association networks and functional enrichment analyses for any sequenced genome of interest. *Nucleic Acids Res*. 2023;51(D1):D638-D646. doi:10.1093/nar/gkac1000

29. Das J, Yu H. HINT: High-quality protein interactomes and their applications in understanding human disease. *BMC Syst Biol*. 2012;6(1):92. doi:10.1186/1752-0509-6-92

30. Orvis J, Gottfried B, Kancherla J, et al. gEAR: Gene Expression Analysis Resource portal for community-driven, multi-omic data exploration. *Nat Methods*. 2021;18(8):843-844. doi:10.1038/s41592-021-01200-9

31. van der Valk WH, van Beelen ESA, Steinhart MR, et al. A single-cell level comparison of human inner ear organoids with the human cochlea and vestibular organs. *Cell Rep*. 2023;42(6):112623. doi:10.1016/j.celrep.2023.112623

32. Bowl MR, Simon MM, Ingham NJ, et al. A large scale hearing loss screen reveals an extensive unexplored genetic landscape for auditory dysfunction. *Nat Commun*. 2017;8(1):886. doi:10.1038/s41467-017-00595-4

33. Groza T, Gomez FL, Mashhadi HH, et al. The International Mouse Phenotyping Consortium: comprehensive knockout phenotyping underpinning the study of human disease. *Nucleic Acids Res*. 2023;51(D1):D1038-D1045. doi:10.1093/nar/gkac972

34. Williams NP, Rodrigues CHM, Truong J, Ascher DB, Holien JK. DockNet: high-throughput protein–protein interface contact prediction. *Bioinformatics*. 2023;39(1). doi:10.1093/bioinformatics/btac797

35. Kozakov D, Hall DR, Xia B, et al. The ClusPro web server for protein-protein docking. *Nat Protoc*. 2017;12(2):255-278. doi:10.1038/nprot.2016.169

36. Wee J, Wei GW. Benchmarking AlphaFold3's protein-protein complex accuracy and machine learning prediction reliability for binding free energy changes upon mutation. *ArXiv*. Published online June 6, 2024.

Table S1.- Rare variants in *TRIOBP*, *CENPJ*, *PLEKHA7* and *SLC41A3* genes found in the occupational noise exposure cohort.

MD Noise Cohort				
**Gene**	***CENPJ***	***CENPJ***	***PLEKHA7***	***SLC41A3***
**Variant**	chr13:24912739T>C	chr13:24879282A>G	chr11:16789275G>A	chr3:126006491T>C
**ID**	rs61739263	rs2275939	rs143911467	rs9833685
**Consequence**	missense_variant	downstream_gene_variant	missense_variant	3_prime_UTR_variant
**Codons**	cAt/cGt	-	Cgc/Tgc	-
**Amino Acid Change**	p.His96Arg	-	p.Arg1060Cys	-
**gnomADg_AF_nfe**	0.000283	0.01	0.000661	0.00147
**CSVS_AF**	0.002	0.003	0.001	0.011
**AF**	0.0588	0.0588	0.0588	0.0588
**OR (CI 95%), *p*-value**	208 (24-829), 0.00202	80 (9.1-313), 0.0136	89 (0.33-350), 0.0002	40 (4.7-157), 0.0065
**n_het**	2	2	2	2
**n_hom**	0	0	0	0
**CADD_PHRED**	0.18	10.01	29.9	0.2
**HL ACMG classification**	Benign	Benign	Uncertain Significance	Benign

* CSVS: Collaborative Spanish Variant Server
** HL ACMG: Hearing Loss American College of Medical Genetics

Table S2.- Rare variants in *PPRC2C* and *NEURL4* genes found in the occupational noise exposure cohort.

MD Noise Cohort Associated to NIHL		
Gene	*PRRC2C*	*NEURL4*
**Variant**	chr1:171536182A>G	chr17:7321200T>A
**ID**	rs138220849	rs145900596
**Consequence**	missense_variant	missense_variant
**Codons**	Atg/Gtg	gAg/gTg
**Amino Acid Change**	p.Met733Val	p.Glu1091Val
**gnomADg_AF_nfe**	4.11E^{-04}	1.76E^{-03}
**CSVS_AF**	0.003	0.012
**AF**	0.0588	0.0588
**OR (CI 95%) *p*-value**	143 (16.6-557), 0.0025	33 (3.9-131), 0.05
**n_het**	1	1
**n_hom**	0	0
**CADD_PHRED**	23	24.5
**HL ACMG classification**	Uncertain Significance	Benign

Table S3.- LGALS8-AS1 lncRNA chr1:236524482A>G variant in the MD noise cohort.

MD Noise Cohort lncRNA

Gene	*LGALS8-AS1*
**Variant**	chr1:236524482A>G
**ID**	rs3820564
**Consequence**	lncRNA
**gnomADg_AF_nfe**	4.31E^{-02}
**CSVS_AF**	0.045
**AF**	0.0588
**OR (CI 95%)** *p*-**value**	1.35 (0.16-5.28), >0.05
**n_het**	2
**n_hom**	0

Table S4.- Differentially accessible peaks in *RFX3* genes on pseudo-Bulk ATACseq MD patients.

**MD - External Controls**						
**Region**	**avg_log2FC**	***p*_val_adj**	**gene_name**	**gene_biotype**	**type**	**distance**
chr9-3397778-3398841	-1.81	1	*RFX3*	protein_coding	gap	0
chr9-3524289-3529143	-1.21	6.52E-^{24}	*RFX3*	protein_coding	utr	0
**MD - Internal Controls**						
chr22-37745682-37746612	-1.07	1.19E-^{13}	*RFX3*	protein_coding	utr	0

Table S5.- *TRIOBP* differentially methylated CpG sites in MD patients.

Chromosome	Position	Gene Name	Average Methylation	Min Value	Max Value
chr22	37711565	*TRIOBP*	97.14	84.85	100
chr22	37711573	*TRIOBP*	94.13	76.47	100
chr22	37711631	*TRIOBP*	69.63	31.25	100
chr22	37711741	*TRIOBP*	97.75	87.50	100
chr22	37711748	*TRIOBP*	91.58	77.78	100
chr22	37729697	*TRIOBP*	95.41	84.62	100

Table S6.- Differentially accessible peaks in *TRIOBP* genes on pseudo-Bulk ATACseq MD patients.

MD - External Controls						
**Region**	**avg_log2FC**	***p*_val_adj**	**gene_name**	**gene_biotype**	**type**	**distance**
chr22-37696386-37697412	-1.50	$2.97x10^{-04}$	*TRIOBP*	protein_coding	utr	0
chr22-37739182-37741840	0.13	1	*TRIOBP*	protein_coding	cds	0
chr22-37745329-37747609	-1.01	1	*TRIOBP*	protein_coding	cds	0
**MD - Internal Controls**						
chr22-37745682-37746612	-0.48	1	*TRIOBP*	protein_coding	cds	0

Table S7.- Splice site prediction for *TRIOBP* chr22:37769343C>T variant in transcripts ENST00000403663 and ENST00000644935.

**Algorithme/Matrix**	HSF Donor site (matrix GT)
**Position**	chr22:37769339
**Sequences**	CGGGCGCAG>CGGGTGCAG
**Variation**	44.03>71.17 (61.64%)
**Signal**	New Donor splice site
**Interpretation**	Activation of a cryptic Donor site. Potential alteration of splicing
**Associated transcripts**	ENST00000403663; ENST00000644935

Table S8.- Creation and deletion of Exonic Splicing Enhancers (ESE) and Exonic Splicing Silencers (ESS) regulatory elements in *TRIOBP* chr22:37769343C>T variant.

Regulatory Elements	Name	Position	Sequence	Status
ESE	ESE_9G8	chr22:37769338	GCGGGC	Site Broken
ESE	ESE_ASFB	chr22:37769339	CGGGCGC	Site Broken
ESS	IIE	chr22:37769340	GGGTGC	Site Created
ESS	Sironi_motif2	chr22:37769341	GGTGCAG	Site Created
ESE	PESE	chr22:37769341	GGCGCAGC	Site Broken
ESE	ESE_ASF	chr22:37769343	CGCAGCA	Site Broken
ESE	ESE_ASFB	chr22:37769343	CGCAGCA	Site Broken

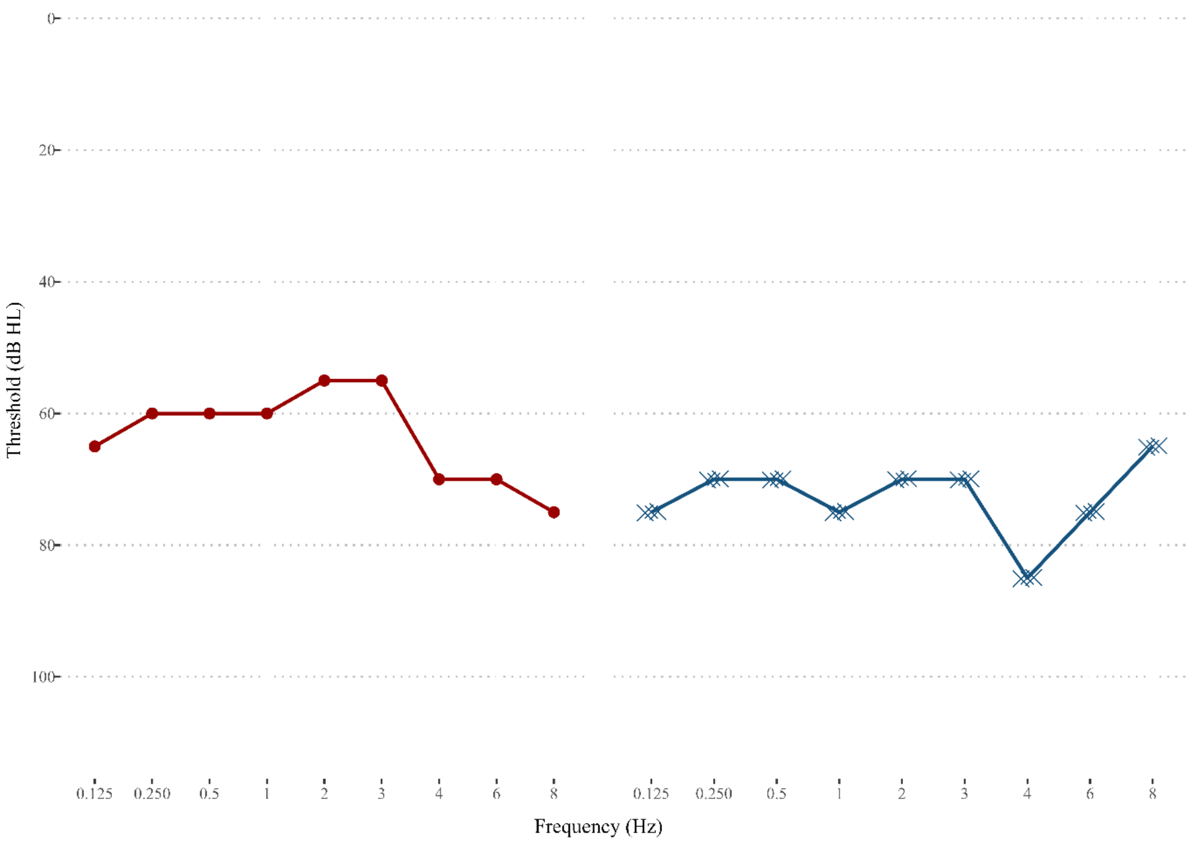

Figure S1.- Family 1 - member II-7 air conducted hearing threshold audiogram with bilateral SNHL. Left ear represented in blue, right in red.

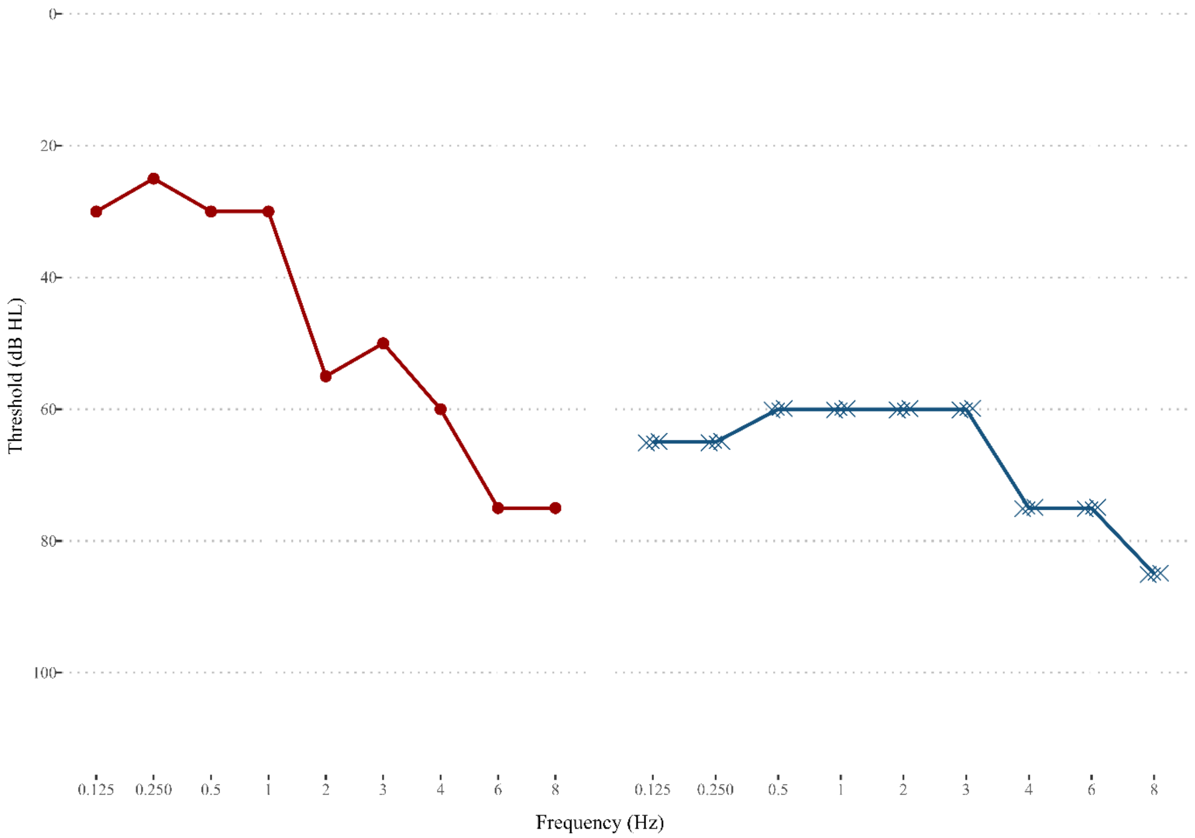

Figure S2.- Meniere Disease individual 3 with occupational noise exposure. Audiogram showing air-conducted pure tone hearing thresholds in their seventh decade of life with bilateral high-frequency hearing loss and SNHL involving all frequencies in the left ear. Right ear is represented in red, left ear in blue.

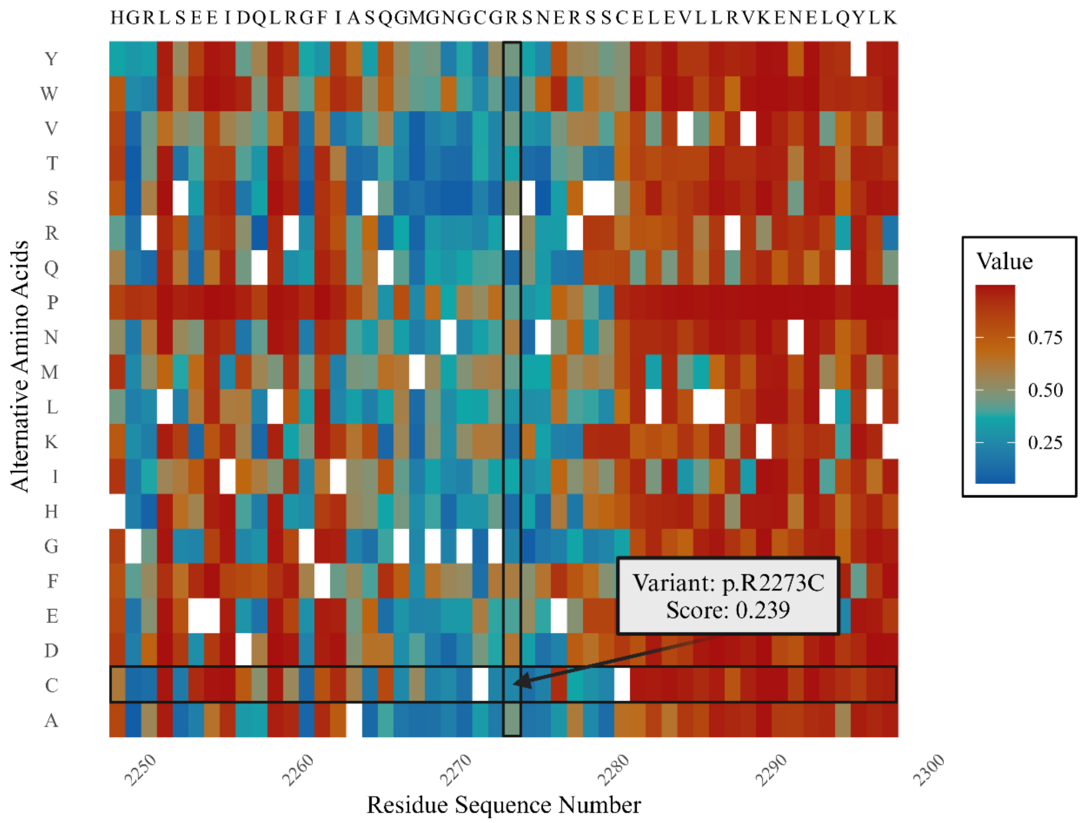

Figure S3.- *TRIOBP* p.R2273C (chr22:37769343C>T) pathogenicity heatmap. AlphaMissense scores *TRIOBP* variant p.R2273C as likely being with a value of 0.239.

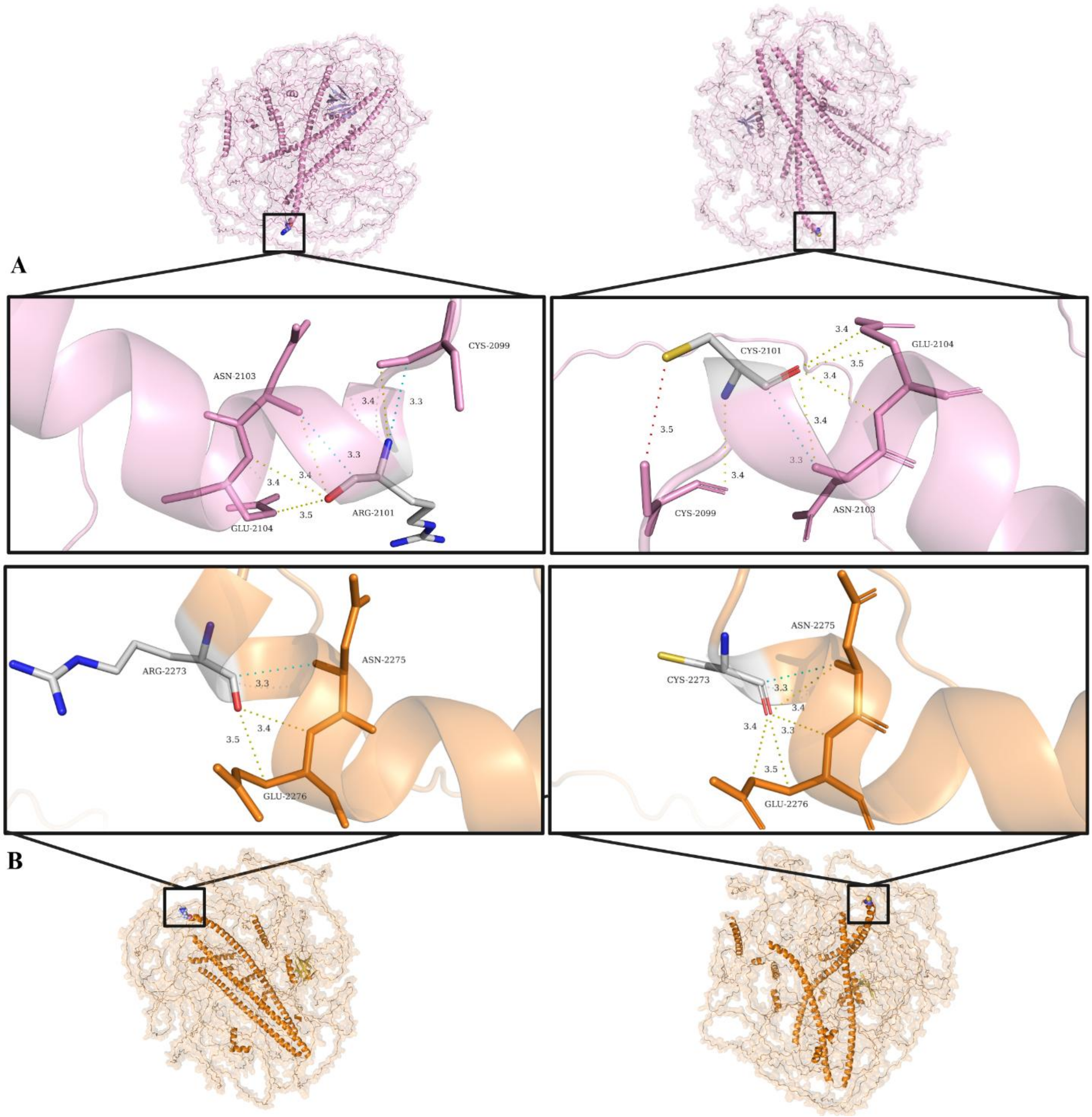

Figure S4.- A) Wild type (left) and mutant (right) TRIOBP-5 protein model, zooming in at the atomic interactions of TRIOBP-5 p.2101. B) Wild type (left) and mutant (right) TRIOBP-6 protein model, zooming in at the atomic interactions of TRIOBP-6 p.2273.

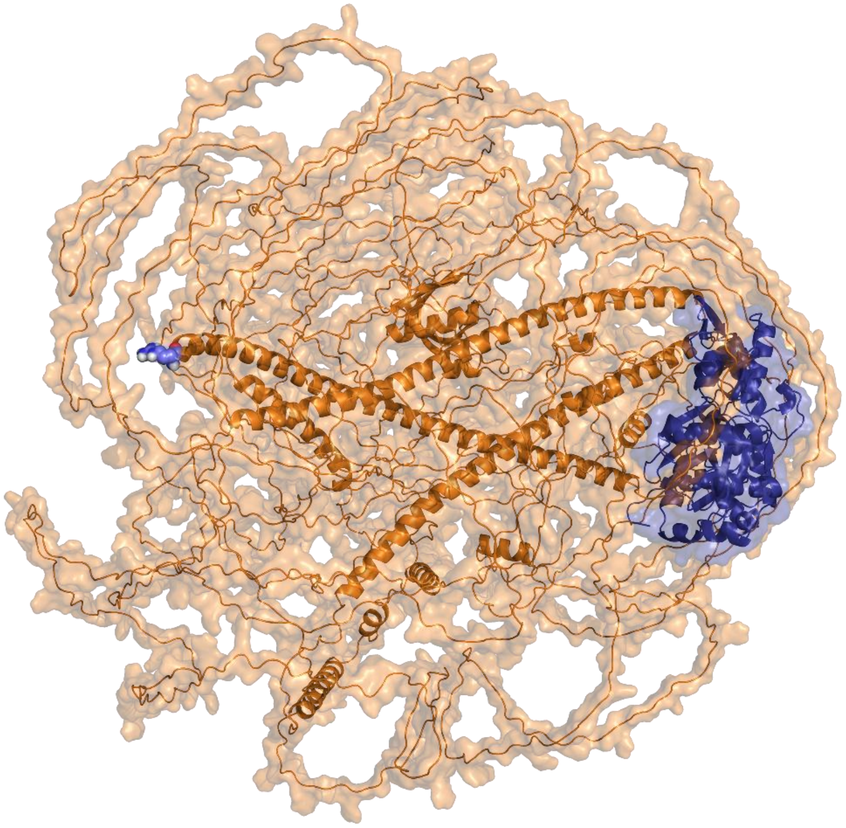

Figure S5.- Docked model between wt *TRIOBP*-6 (orange) and *ACTB* (blue). Variant p.2273R was not predicted to interact with *ACTB*.

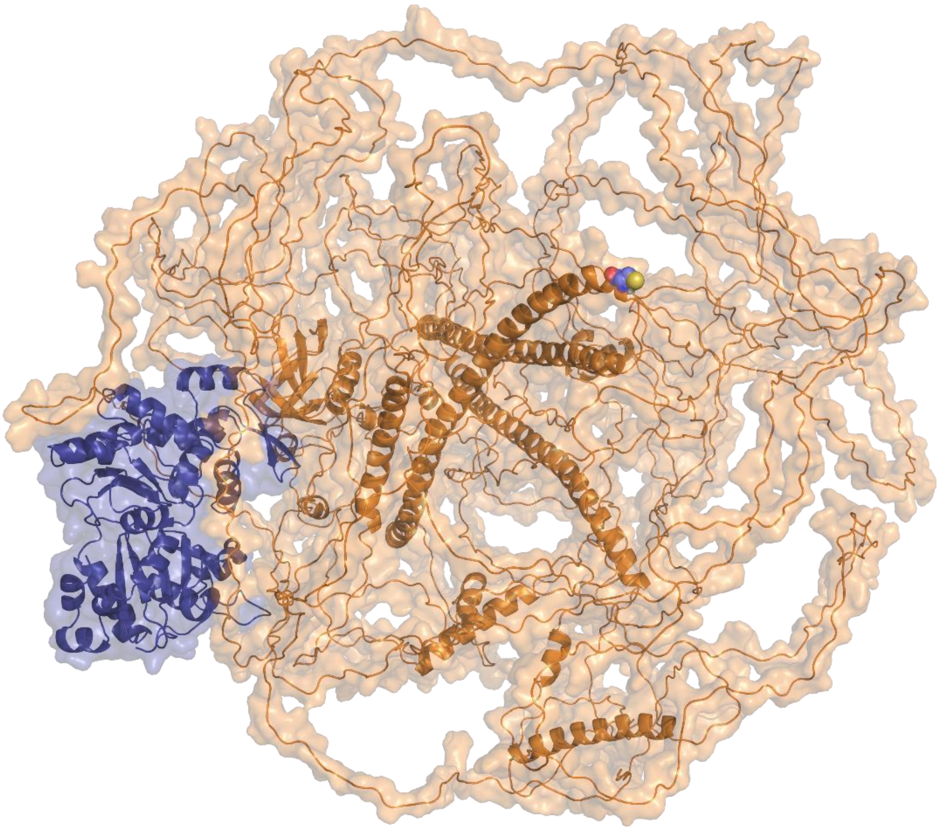

Figure S6.- Docked model between mt *TRIOBP*-6 (orange) and *ACTB* (blue). Variant p.2273C was not predicted to interact with *ACTB*.

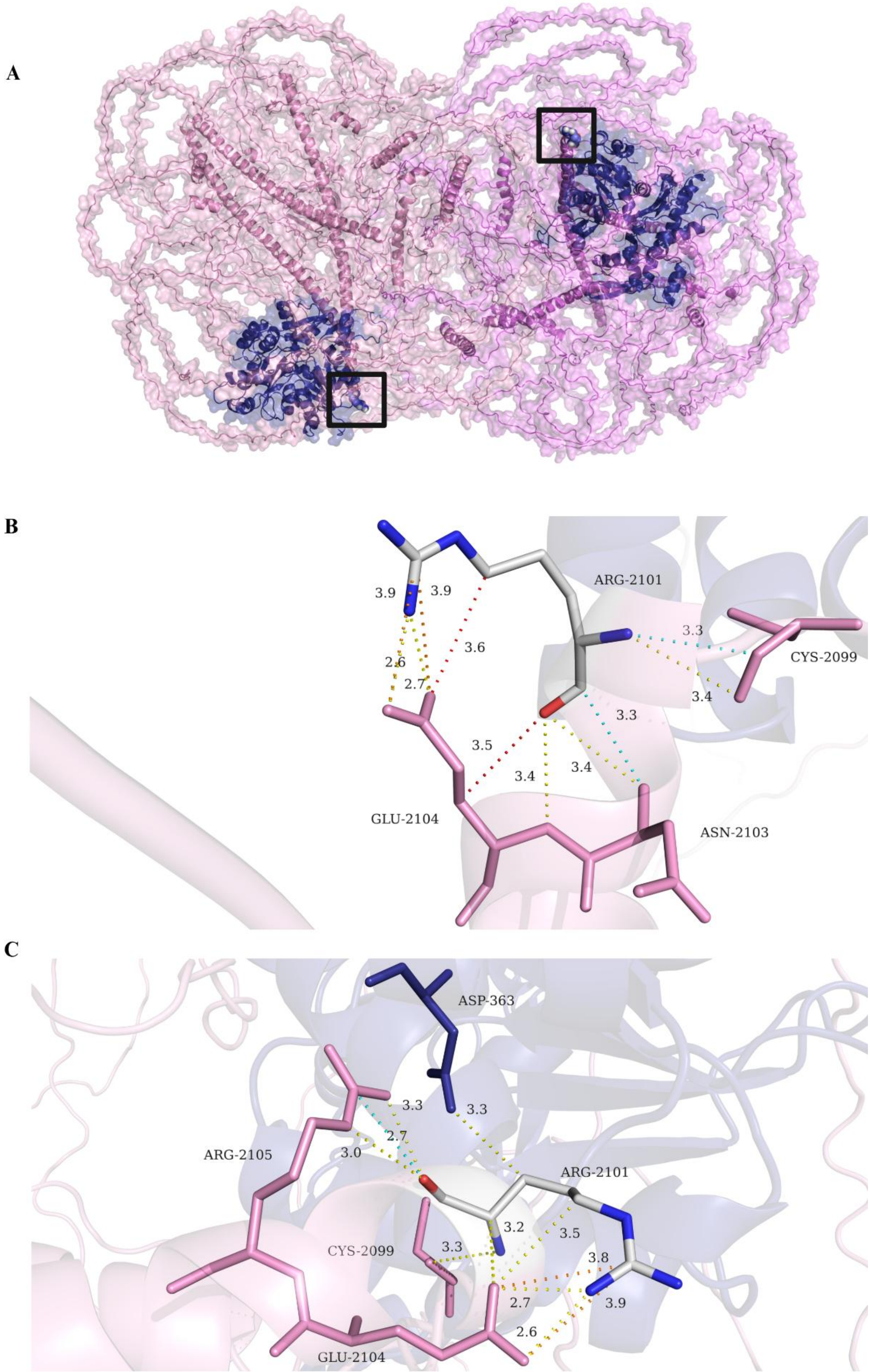

Figure S7.- A) Wild type *TRIOBP*-5 dimer docked model to *ACTB* chain C. B) Polar contacts of wt *TRIOBP*-5 dimer docked to *ACTB* chain A. B) Polar contacts of wt *TRIOBP*-5 dimer docked to *ACTB* chain C.

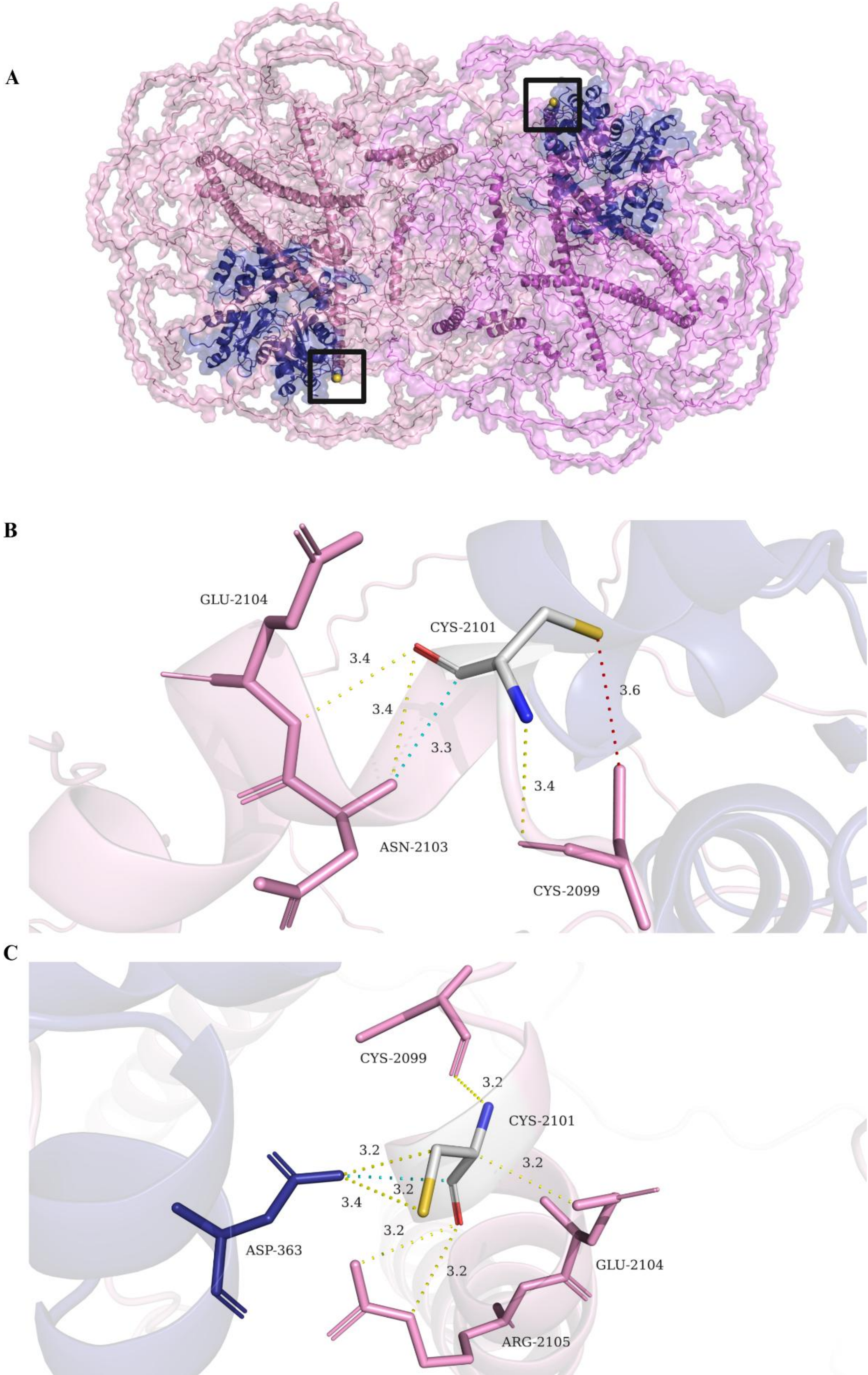

Figure S8.- A) Mutant *TRIOBP*-5 dimer docked model to *ACTB* chain C. B) Polar contacts of mutant *TRIOBP*-5 dimer docked to *ACTB* chain A. B) Polar contacts of mutant *TRIOBP*-5 dimer docked to *ACTB* chain C.

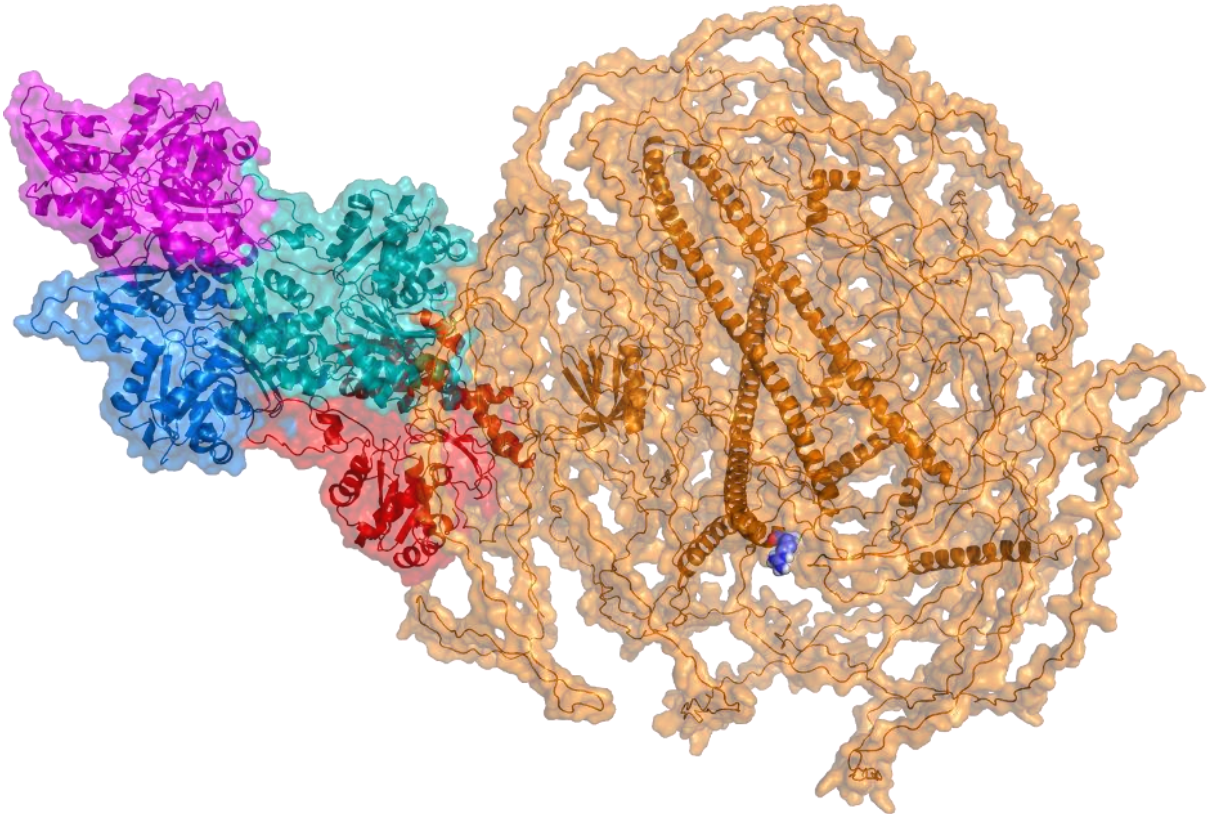

Figure S9.- Docked model between wt *TRIOBP*-6 (orange) and F-Actin (magenta, cyan, blue and red). Variant p.2273R was not predicted to interact with F-Actin.

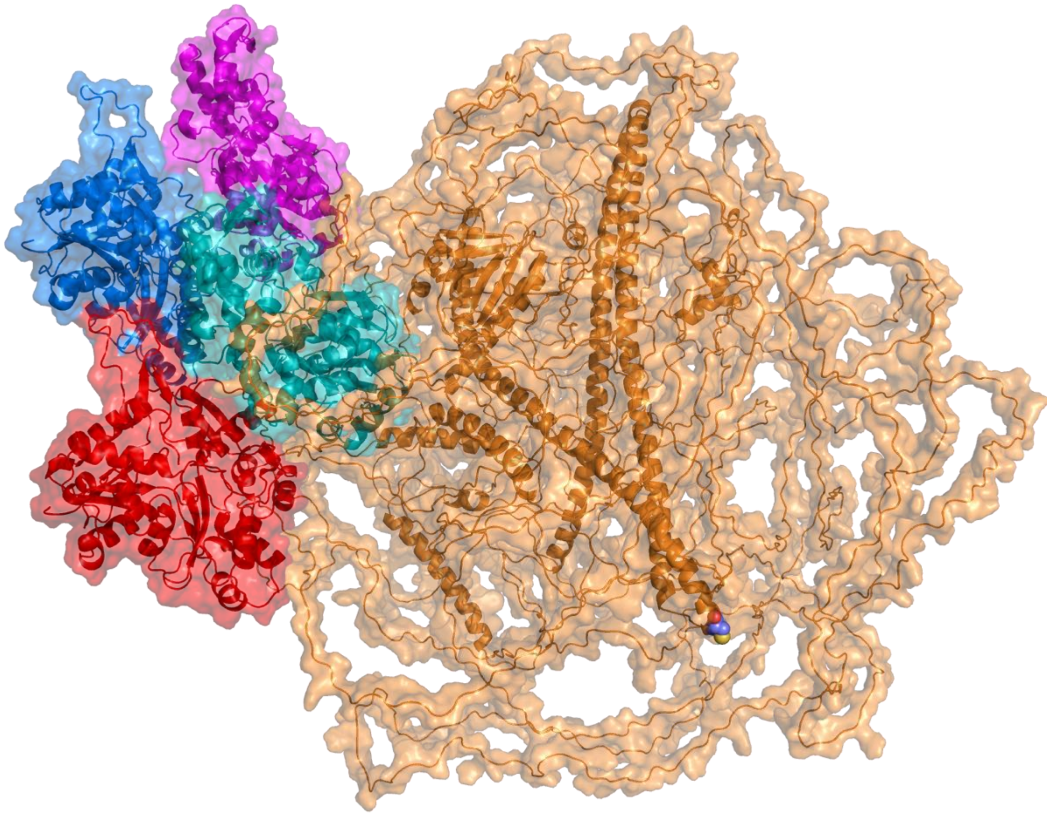

Figure S10.- Docked model between mt *TRIOBP*-6 (orange) and F-Actin (magenta, cyan, blue and red). Variant p.2273C was not predicted to interact with F-Actin.

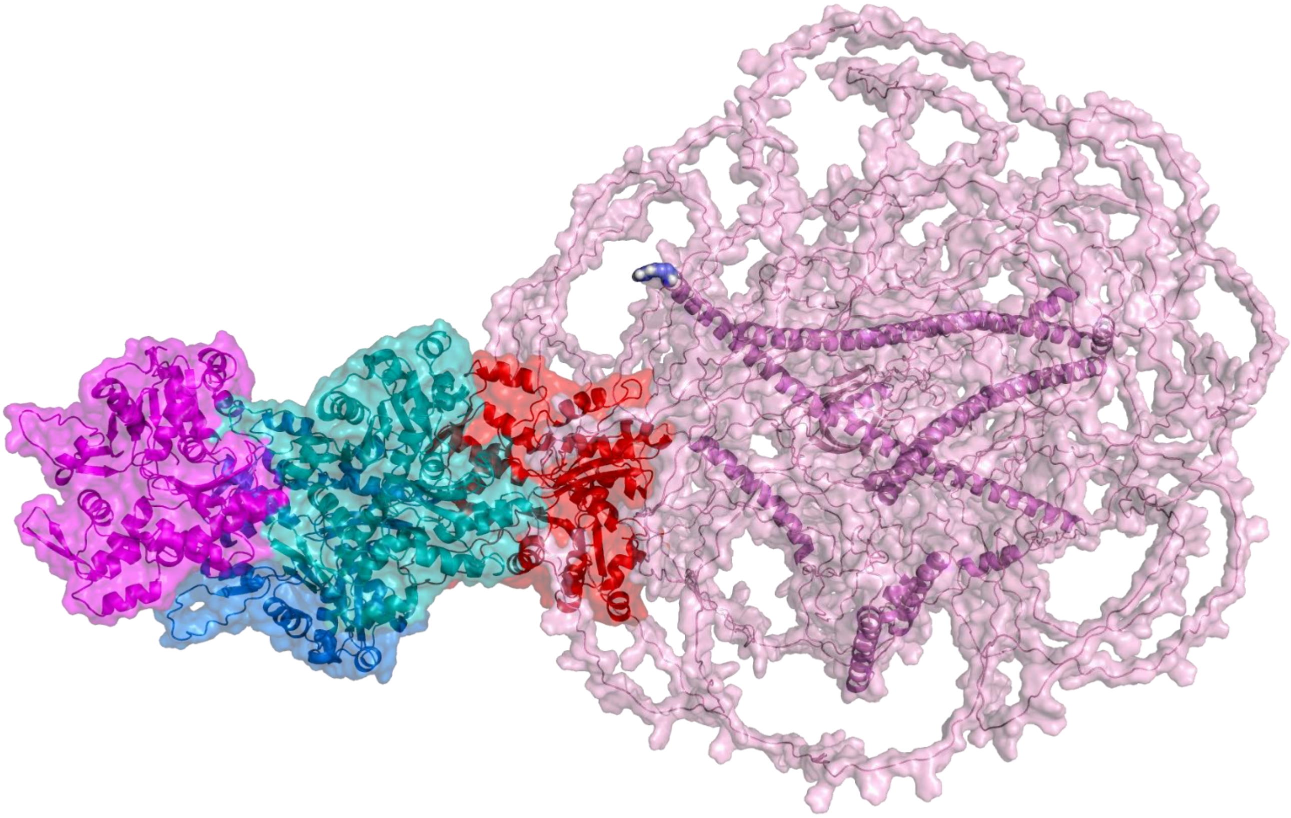

Figure S11.- Docked model between wt *TRIOBP*-5 (orange) and F-Actin (magenta, cyan, blue and red). Variant p.2273R was not predicted to interact with F-Actin.

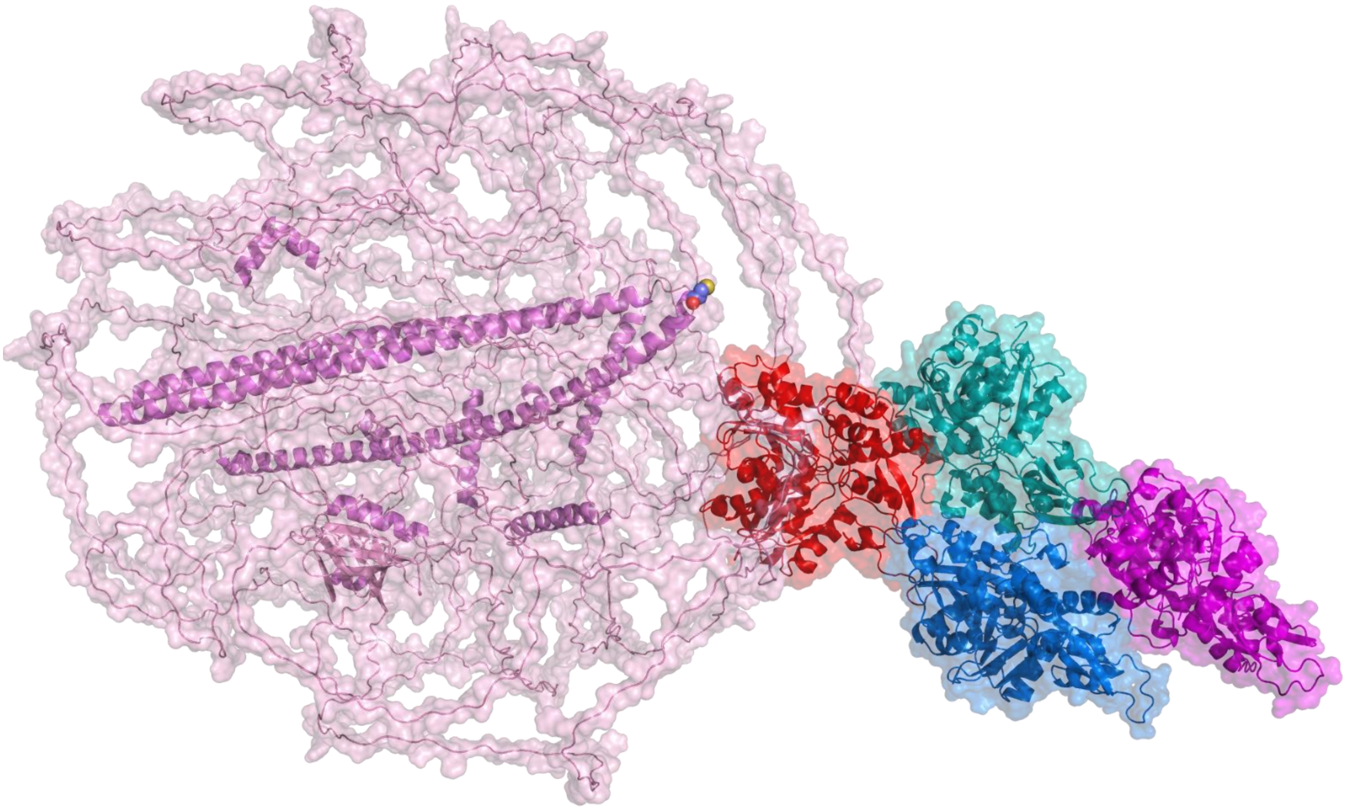

Figure S12.- Docked model between mt *TRIOBP*-5 (orange) and F-Actin (magenta, cyan, blue and red). Variant p.2273C was not predicted to interact with F-Actin.

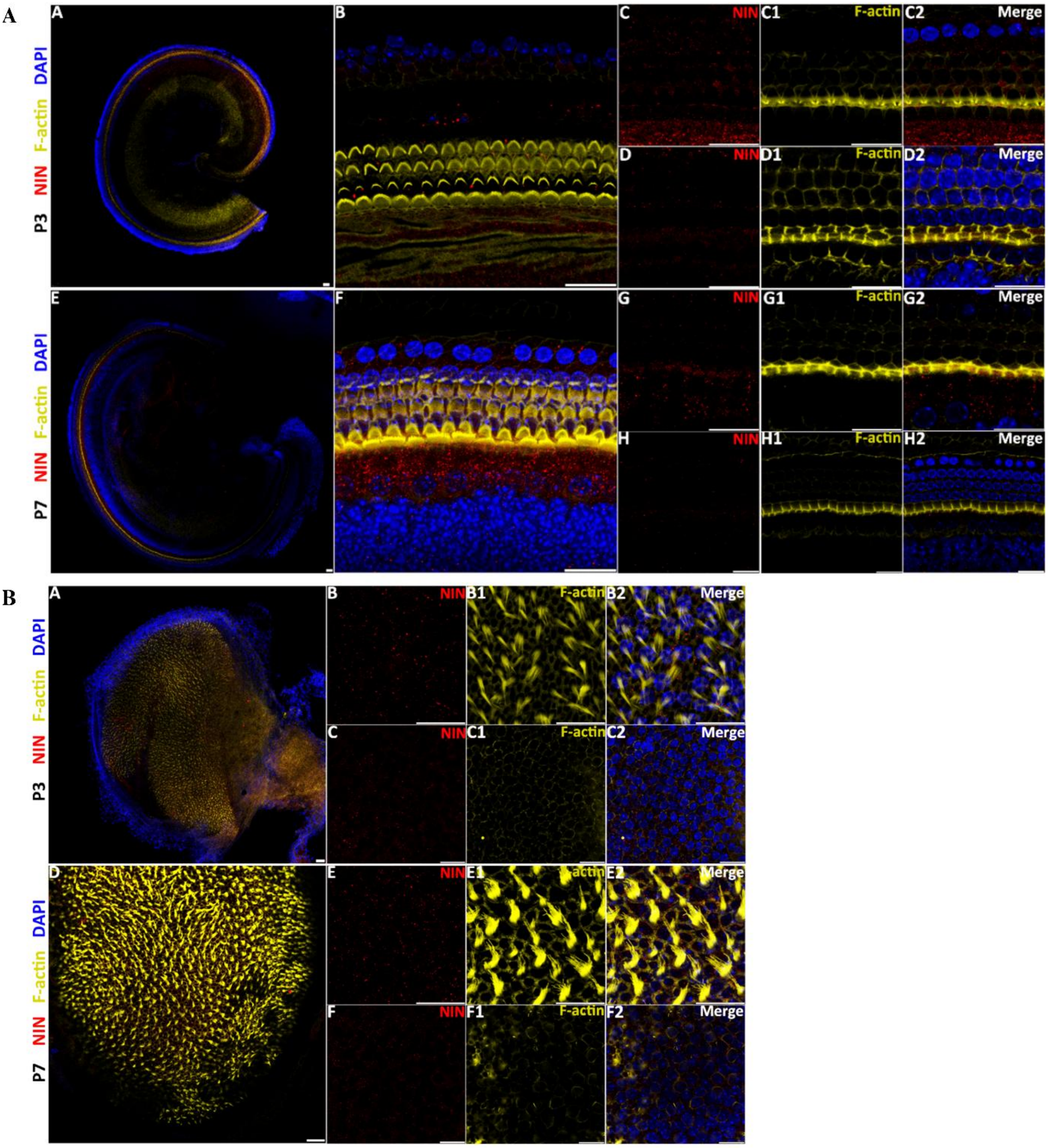

Figure S13.- Ninein protein (*NIN* gene) expression in the cochlear basilar membrane and vestibule. A) A, Postnatal day 3 (P3) mouse cochlear basilar membrane, apical and middle turn. B, Magnified overlay image of A. C, C1, C2, Layers above the hair cells nuclei. D, D1, D2, Nuclear layers of hair cells. E, Postnatal day 7 (P7) mouse cochlear basilar membrane, apical and middle. F, Magnified overlay image of E. G, G1, G2, Layers above the of hair cells nuclei. H, H1, H2, Nuclear layers of hair cells. Bar: 20µm. B) A, Postnatal day 3 (P3) mouse saccule. B, B1, B2, Stereocilia layer of vestibular cells. C, C1, C2, Nuclei layer of vestibular cells. D, Postnatal day 7 (P7) mouse utricle. E, E1, E2, Stereocilia layer of vestibular cells. F, F1, F2, Nuclei layer of vestibular cells. Bar: 20µm.

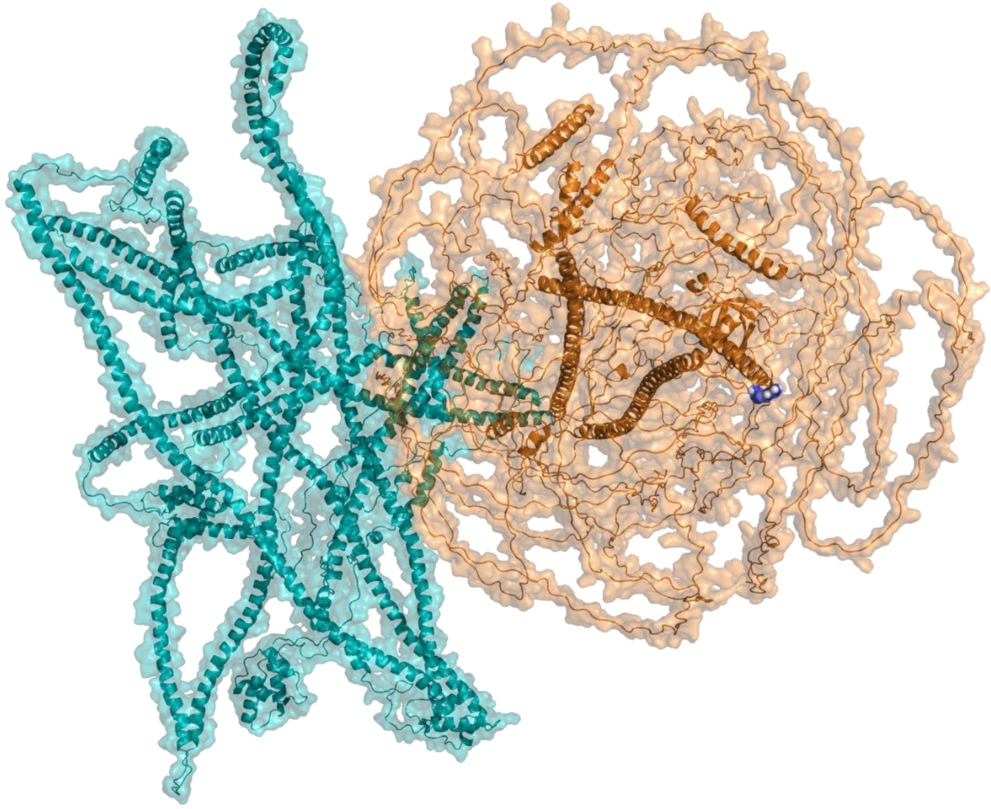

Figure S14.- Docked model between wt *TRIOBP*-6 (orange) and *NIN* (cyan). Wt p.2273R was not predicted to interact with *NIN*.

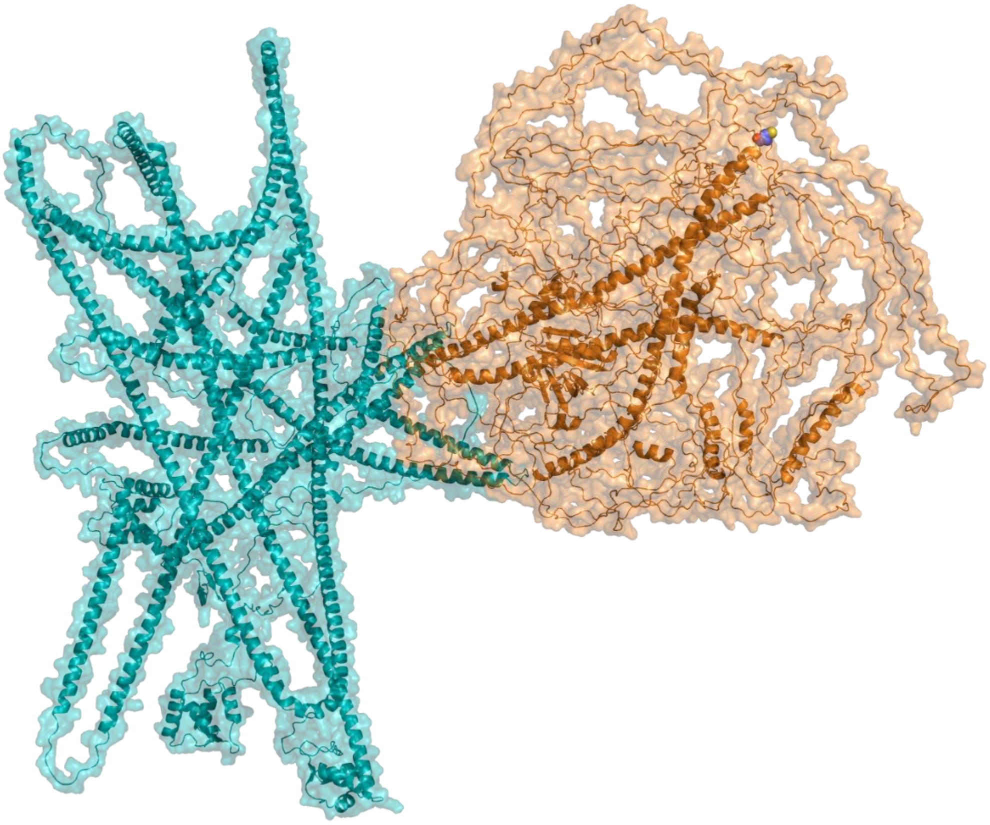

Figure S15.- Docked model between mt *TRIOBP*-6 (orange) and *NIN* (cyan). Variant p.2273C was not predicted to interact with *NIN*.
